# Diversity and Baits Preference of Flower Flies (Diptera: Syrphidae) Collected Using Van Someren-Rydon Traps in the Colombian Andean-Amazon Piedmont During Two Rainy Seasons

**DOI:** 10.1007/s13744-025-01260-y

**Published:** 2025-03-27

**Authors:** Henry Mauricio Parada-Marin, Augusto León Montoya, Yardany Ramos-Pastrana

**Affiliations:** 1 Grupo de Investigación en Entomología, Universidad de La Amazonia (GIEUA), Florencia, Caquetá Colombia; 2 Grupo de Entomología, Universidad de Antioquia (GEUA), Medellín, Antioquia Colombia

**Keywords:** Larval trophic groups, Neotropical Syrphid flies, New records, Sampling Method, Seasonality, Tropical rainforests

## Abstract

Thirty percent of the flower flies (Diptera: Syrphidae) diversity is concentrated in the Neotropics, with few studies in the Amazonian rainforests. We evaluated diversity, phenology and bait preference of syrphids in three lowland habitats (FE = Forest Edge, DSF = Dense Secondary Forest and AFS = Agroforestry System) in La Avispa Nature and Ecotourism Reserve in Caquetá, Colombia, during two rainy seasons (high rainfall intensity, HIR and low rainfall intensity, LIR). Adults were collected using Van Someren-Rydon traps (VSRTs) baited with fermented fruit, decomposed shrimp or decomposed fish. We estimated alpha and beta diversity components within and between habitats. Phenology was compared between rainy seasons. A total of 1,379 syrphids specimens were collected, belonging to 59 species (13 genera). DSF had the highest abundance and richness (*n* = 812; 43 species). Syrphid abundance was 8.9 times higher (*n* = 1.240) during HIR, while richness was 1.8 times higher (68 species) than LIR. *Copestylum* was the most abundant and diverse genus (*n* = 1,225; 88.8% of all specimens collected). The VSRTs showed high sampling completeness (≥ 90%), suggesting that this methodology is useful and effective for rapid Syrphids inventories. Species replacement was the dominant component of beta diversity, indicating that each habitat contributes with unique species, providing functions for sustainability and ecosystem balance, where connectivity is essential to ensure the provision of ecosystem services in undergoing ecological restoration areas, such as the tropical rainforest studied here.

**Supplementary Information:**

The online version contains supplementary material available at 10.1007/s13744-025-01260-y.

## Introduction

In Neotropical ecosystems, flower flies (Diptera: Syrphidae) are key components, because they provide ecosystem services in most links of food chain, contributing not only to pollination as adults but also as crop pest controllers, preying mosquito vectors of diseases as well as playing a decisive role as organic matter recyclers and soil formers in their immature stages (Rotheray & Gilbert 2011; Montoya et al. 2021). A relevant aspect in the study of Syrphidae as bioindicator groups is the identification of effective and standardized methods that allow to relate rainy seasonality with habitat preference of each larval trophic group (Ángel Villarreal et al. 2021; Gittings et al. 2006; Marcos-García et al. 2012; Naderloo and Pashaei 2014; Montoya et al. 2021).

The tropical humid forests (bh-T) that make up the Colombian Amazon comprise an area of 415.000 km^2^ (Etter 1993), characterized by high plant heterogeneity, structural and environmental complexity, which harbors about 50% of the described plant species (Gentry 1993). Today, the Amazon region forms small “fragmented forest islands” with patchy mosaics of primary vegetation, surrounded by “grassland oceans” (Smith 2021). Despite this, the Amazon region offers ideal environmental characteristics to harbor a high diversity and uniqueness in flower fly fauna (Reemer 2010, 2016; Miranda et al. 2014, 2017a, 2017b; Miranda et al. 2014, 2020, 2022; Parada-Marín et al. 2021; Montoya et al. 2022; Parada-Marin et al. 2024), playing a determining role in the dynamics of links in the food webs in these singular ecosystems (Montoya et al. 2021). Although no Syrphid species lists have been made in the Colombian Amazon region (Gutiérrez et al. 2006; Restrepo-Ortiz and Carrejo 2009; Montoya et al. 2012; Montoya 2016), in recent years, distributional and taxonomic research has been conducted on the group (Parada-Marín et al. 2021; Montoya et al. 2022).

The structure of population assemblages of insects varies in space and time (Noriega et al. 2021), consequently, evaluating the diversity variation and phenology along an environmental gradient is fundamental to identifying the degree of habitat recovery undergoing ecological restoration, where the structure and composition of trophic functional groups give signs of environmental sustainability and balance (Sommaggio 1999; Montoya et al. 2021).

Studies assessing Syrphidae biodiversity have mainly employed Malaise traps, colored pan-traps, and/or entomological nets for collecting adults. Immatures have been reared and analyzed according to their functional groups (mainly phytophages, saprophages or zoophages), finding out that sampling effectiveness can be dependent on syrphid trophic or functional group, climatic season and environmental characteristics (Rotheray et al. 2007; Reemer 2010; Marcos-García et al. 2012; Sommaggio and Burgio 2014; Souza et al. 2014; Ángel Villarreal et al. 2021; Montoya et al. 2021).

The baited Van Someren-Rydon Traps (VSRTs) have been used mainly in studies of diversity and distribution of butterflies (Lepidoptera) (Rydon 1964; Villarreal et al. 2004), obtaining good results in different vegetation covers (Brown and Freitas 2002; Freitas et al. 2014; Casas-Pinilla et al. 2017; Álvarez et al. 2021). These traps have also been employed in studies of Diptera, managing to be very effective in Synanthropic studies of Calliphoridae (Montoya et al. 2009; Pinilla et al. 2012; Ramos-Pastrana et al. 2022), Muscidae (Ramos-Pastrana et al. 2022) and Sarcophagidae (Pinilla et al. 2012; Yepes-Gaurisas et al. 2013). However, studies evaluating the effectiveness and bait preference by Syrphids are scarce in the available scientific literature (Camargo et al. 2023).

Therefore, the objective of this study was to determine the variation in diversity and abundance of Syrphidae throughout two rainy seasons (high and low-intensity rainfall), as well as structure and composition (beta diversity within and between habitats) in three habitat types (Dense Secondary Forest, Forest Edge and Agroforestry System) in La Avispa Nature and Ecotourism Reserve in the Colombian Andean-Amazon piedmont. In addition, we aimed to evaluate the effectiveness and bait preference of Van Someren-Rydon in collecting Syrphidae adults in the three habitats and during the two rainy seasons.

## Materials and Methods

### Sampling Areas

**Fig. 1:**
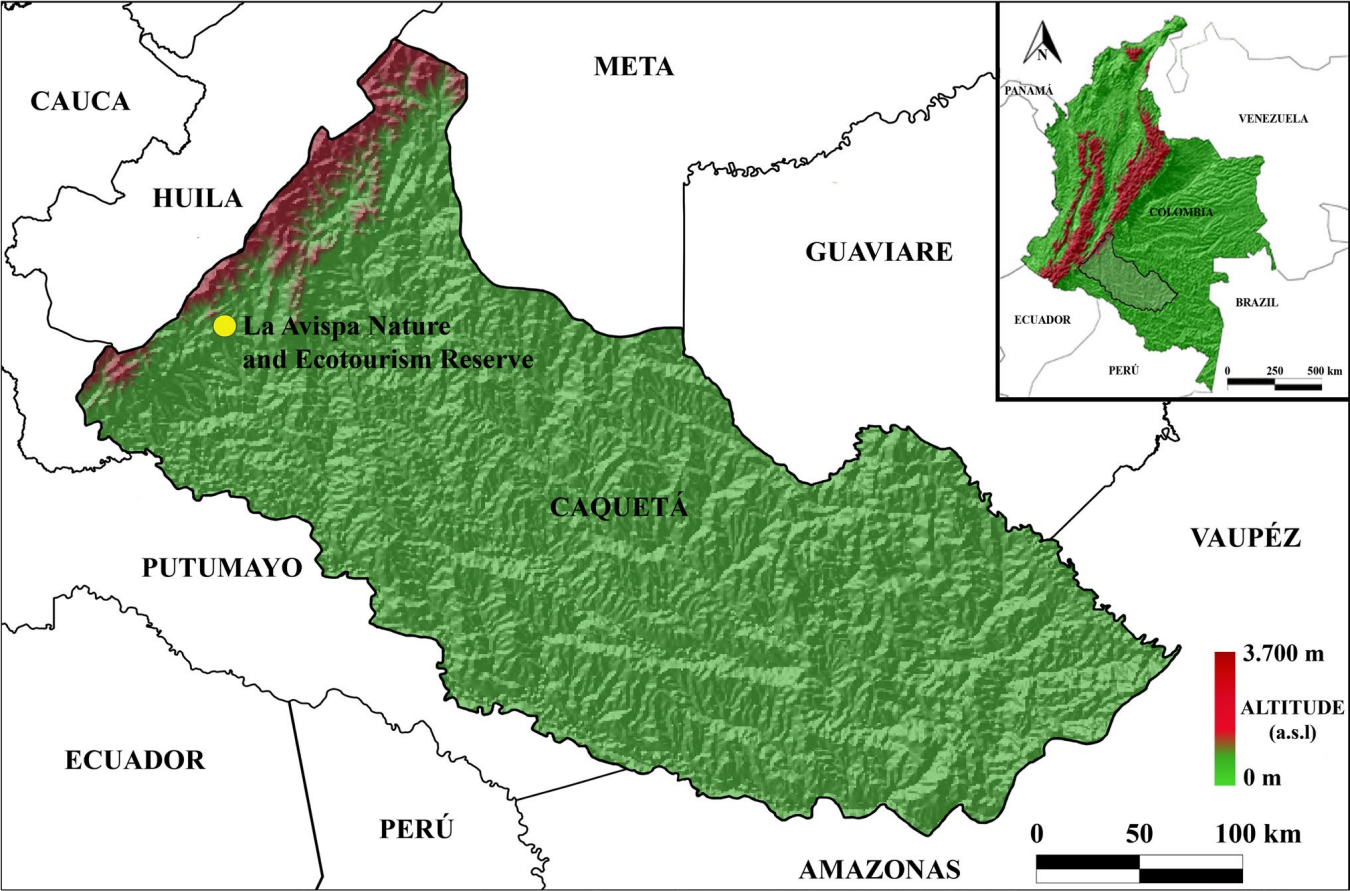
Geographic location of La Avispa Nature and Ecotourism Reserve, Caquetá, Colombia (QGIS Development Team 2023)

The study was carried out in the La Avispa Nature and Ecotourism Reserve (Reserva Natural y Ecoturística La Avispa) (1°37'N, 75°40'W, average altitude of 430 m.a.s.l.), that is situated in the township of Santo Domingo, west of the municipality of Florencia, Caquetá, in the Colombian Amazon rainforest (Fig. 1). The reserve covers 232 hectares, located in the transition zone between the Andean and Amazonian regions (Andean-Amazon piedmont), comprising a wide variety of ecosystems, making it one of the most diverse areas in Colombia (Telis 2016; Silva-Olaya et al. 2022), considered a biodiversity hotspot for conservation priorities (Myers et al. 2000). The syrphid fly composition in the reserve was evaluated in three lowland habitats with different plant diversity: Forest Edge (FE), Dense Secondary Forest (DSF), and Agroforestry System (AFS) (see the Supplementary Material for details; Table S1). The climatic conditions in the area is characterized by an average temperature of 25 °C, average relative humidity of 83% and annual precipitation of 3,840 mm. The precipitation is bimodal, with low-intensity rainfall (LIR) occurring from September to March, while high-intensity rainfall (HIR) covers April to August (IGAC 2010; IDEAM 2015; Ramos-Pastrana et al. 2021).

### Sampling of Adult Flower Flies by Van Someren-Rydon Trap and Baits Preference

In each habitat, adult flower flies were collected using Van Someren-Rydon traps (VSRT) installed in nine sampling points. The VSRT consisted of a cylindrical mesh, sealed at the top and opened at the bottom (Freitas et al. 2014), placed spatially separated by at least 50 m from each other and located 1.5 m above ground. Each trap was georeferenced with the help of a Garmin GPS (model 64 s) using the WGS84 coordinates system. Three baits were tested: “shrimp”, “fruit” and “fish”. The “shrimp” bait consisted of decomposing shrimp [*Protrachypene precipua* (Burkenroad, 1934)]. The “fruit” bait was composed of a mixture of fermented banana (*Musa* sp.) and pawpaw fruits (*Carica pawpaw* L.), while the “fish” bait consisted of decomposing *Astyanax bimaculatus* (L). The fresh ingredients of baits were obtained in the local market and then placed in separate containers to decompose for three days before going to the field. Each trap was set up with one bait (250 g) and three replicates in each habitat.

A Kruskal–Wallis test (α = 0.05) was performed using the *dunn.test* package (Dinno 2024) in Rstudio software version 4.3.1 to evaluate if there was a significant difference between baits (R Core Team 2023). Since the results of Camargo (2023) suggest that VSRTs baited with fermented fruit can be useful for capturing hoverflies, this bait was used as a control in the statistical analysis.

### Identification, Counting of Captured Specimens and Larva Trophic Groups of Flower Flies

The captured specimens were preserved in 95% ethanol and a reference collection was prepared for identification, following the methodology proposed by Márquez (2005). Species were determined using the taxonomic keys of Thompson (2006), Thompson et al. (2010), Reemer (2010, 2016), Ricarte et al. (2015), Miranda (2017a) and Montoya et al. (2022). The species confirmation was performed with the extraction of male genitalia following the methodology proposed by Montoya et al. (2022) and by comparison with reference specimens deposited in the Laboratorio de Entomología Universidad de la Amazonia -LEUA (Florencia, Caquetá, Colombia) and Colección Entomológica Universidad de Antioquia -CEUA (Medellín, Antioquia, Colombia).

Adult flower flies of each species or morphospecies captured per bait were counted to determine the following faunal parameters: Abundance; Absolute abundance (A.A.); Relative abundance (R.A); Total abundance (N); Relative frequency (R.F.%) and richness (S) following the definitions proposed by Crupi (2019). Flower flies were considered a dominant species when up to 20 specimens were captured per bait in each habitat and rainy season. The trophic categories were assigned based on food resource preferences of larvae of each genus: saprophagous, xilosaprophagous or zoophagous, including also microhabitat specificity: terrestrial or aquatic, following the criteria for functional trophic groups proposed by Montoya et al (2021).

## Data Analysis

### Flower Fly Phenology Analysis and Structure of the Community Assemblages

The collections of adult flower flies were conducted from February to May of 2022, encompassing two rainy seasons: high-intensity rainfall (HIR) and low-intensity rainfall (LIR). A total of nine non-consecutive sampling days were conducted for each season: LIR = 18, 19, 20, 25, 26, 27 February, 4, 5, 6 March 2022, and HIR = 29, 30 April, 1, 7, 8, 9, 13, 14, 15 May 2022, with samples collected in the morning and afternoon on each sampling day.

The influence of seasonality on diversity and abundance was analyzed using a phenology plot, allowing us to appreciate the relationship between the rainy seasons (HIR and LIR) and the diversity in each habitat. Only flower fly species with ≥ 10 individuals (throughout the samplings) were considered for phenology (shifts over time) analysis, to which the habitats and months of occurrence were analyzed, during both rainy seasons. Additionally, rank-abundance curves were elaborated, these curves allow us to identify differences in the structure between communities, identify dominant and rare species, as well as their abundance equity and sequence (Whittaker 1972; Magurran 1988). The curves were plotted using GraphPad Prism software version 10.0.3, considering the sequence of absolute abundances of each species per habitat and season.

### Flower Fly Species Diversity

Species diversity connects species richness and their relative abundance and its metrics can be applied at different scales (Magurran 2021). We used alpha diversity (species composition within a habitat) and beta diversity (differences in species composition among habitats) to capture the flower fly diversity. The alpha diversity was evaluated using Hill numbers ^q^D. This diversity index allows to obtain a broad perspective of species richness, species rarity, dominance and evenness (the relative proportion of each species within the sampled assemblage) (Hill 1973; Moreno 2001). We used three measures of Hill numbers of order q: species richness (q0), Shannon diversity in its exponential form (q1) and Simpson’s inverse index (q2) as flower fly diversity indices (Chiu and Chao 2020). Hill numbers were calculated for total sampling period (covering the two rainy seasons) and each habitat per rainy sampling season using the free program SpadeR Online (Chao et al. 2015), considering the distribution of species recorded in each habitat, the observed species richness (q0) and the most abundant species (q2) (Chao et al. 2015).

Sampling effort and expected richness were assessed by interpolation and extrapolation curves based on sampling-coverage (i.e., the proportion of individuals that belong to observed species in a sample), using the sample-size method, with a bootstrap of 200 replicates, based on abundances (Chao et al. 2014). The analyses were differentiated by rainy season and habitats based on Hill’s numbers, for which q0 (observed richness), q1 (Shannon’s exponential based on shared species) and q2 were considered and curves obtained using the free software iNEXT Online (Chao et al. 2016). Comparisons of the compositional flower fly communities were made by habitat pairs, using the Jaccard index and considering two components of the beta diversity: species turnover and nestedness, as suggested by Baselga (2010) and Montoya et al. (2021). The beta diversity was performed using the *betapart* package in R version 1.6 (Baselga et al. 2023). In addition, a Venn diagram was made to characterize the structure in terms of the number of exclusive and shared species for each habitat.

### Non-metric Multidimensional Scaling (nMDS) and PERMANOVA

Overall patterns of similarity, concerning species composition and relative abundances between habitats and baits were explored by a non-metric multidimensional scaling (nMDS) analysis using the Bray–Curtis abundance-based similarity index (Bray and Curtis 1957) and the Jaccard presence-absence (incidence) based similarity index (Jaccard 1908). Similarity differences were assessed using permutational multivariate analysis of variance (PERMANOVA) with 9,999 permutations of residuals, as a non-parametric variant to multivariate analyses of Bray–Curtis and Jaccard values, for habitats and baits (Bui et al. 2010; Musthafa et al. 2021). The nMDS allows representing the degree of similarity of habitats and baits in terms of abundances and species composition in a two-dimensional space (Musthafa et al. 2021). To decrease the effect of dominant species and give greater weight to rare species whose abundances were low, data were standardized by applying square root to absolute abundances, for subsequent analysis using PRIMER v7 software (Clarke and Gorley 2015). In addition, a bar diagram was made to show the proportions of effectiveness and bait preference in each habitat, using the *ggplot2* package (Wickham 2016) in the Rstudio software version 4.3.1 (R Core Team 2023).

The composition of the flower flies was also analyzed considering the abundance and richness of exclusive and shared species between each habitat using a linear discriminant analysis, which allows the recognition of similarity patterns (Fisher 1936). Linear discriminant analysis was performed considering the number of genera, species and individuals collected about bait, the larval trophic category and habitat. The analysis was performed using the *MASS* package (Venables and Ripley 2002) in Rstudio software version 4.3.1 (R Core Team 2023).

## Results

### Abundance and Diversity of Flower Flies

We captured a total of 1,379 adult flower flies considering all Van Someren-Rydon traps installed in the three habitats from February to May of 2022. Fifty-nine valid species and thirteen morphospecies in 13 genera were collected, depicted in two subfamilies: Eristalinae (*n* = 1275, 5 genera and 54 species) and Syrphinae (*n* = 104, 8 genera and 18 species) (Table 1; Supplementary Material: Fig. S1). The term morphospecies was used when the identification did not fit with valid species descriptions or available taxonomic keys, and some could be undescribed species.

**Table 1:** Number of specimens per species or morphospecies of adult flower flies (Diptera: Syrphidae), their abundance (total number of counted adults) and larval trophic category, captured by three different baits (decomposing shrimp, fermented fruit or decomposing fish) using Van Someren-Rydon traps in three habitats (Forest Edge, Dense Secondary Forest and Agroforestry System) of La Avispa Nature and Ecotourism Reserve, Caquetá, Colombia, during two rainy seasons (High-intensity and low-intensity rainfalls) from February to May of 2022

Flower fly Subfamily / Species	Larval trophic category	High-intensity rainfall									Low-intensity rainfall									A.A	R.A	R.F.%
		Forest Edge			Dense Secondary Forest			Agroforestry System			Forest Edge			Dense Secondary Forest			Agroforestry System					
		Shrimp	Fruit	Fish	Shrimp	Fruit	Fish	Shrimp	Fruit	Fish	Shrimp	Fruit	Fish	Shrimp	Fruit	Fish	Shrimp	Fruit	Fish			
**Eristalinae**																						
Copestylum alcedoides**	Aq.Sap	-	-	-	-	-	2	-	-	-	-	-	-	-	-	-	-	1	-	3	0.002	0.22
*Copestylum araceorum*	Aq.Sap	2	-	3	10	-	14	-	-	4	-	-	1	-	-	1	-	-	-	35	0.025	2.54
Copestylum bassleri**	Aq.Sap	-	-	1	2	-	7	1	-	11	-	-	1	-	-	-	-	-	-	23	0.017	1.67
Copestylum brevivitatum*	Aq.Sap	-	-	-	2	-	1	-	-	-	-	-	-	-	-	-	-	-	-	3	0.002	0.22
Copestylum cinctiventre*	Aq.Sap	1	-	1	-	-	3	2	-	2	-	-	-	-	-	1	-	-	-	10	0.007	0.73
Copestylum circumdatum*	Aq.Sap	-	-	1	-	-	-	-	-	1	-	-	-	-	-	-	-	-	-	2	0.001	0.15
Copestylum contumax*	Aq.Sap	-	-	-	-	-	2	-	-	-	1	-	-	1	-	-	-	-	-	4	0.003	0.29
Copestylum cyanoproctum**	Aq.Sap	1	-	1	6	-	23	2	-	3	-	-	1	-	-	1	-	-	-	38	0.028	2.76
Copestylum cyclops**	Aq.Sap	-	-	1	-	-	2	-	-	-	-	-	-	-	-	-	-	-	-	3	0.002	0.22
Copestylum discale**	Aq.Sap	-	-	1	-	-	-	-	-	-	-	-	-	-	-	-	-	-	-	1	0.001	0.07
*Copestylum enriquei*	Aq.Sap	-	-	-	-	-	10	-	-	-	-	-	-	-	-	-	-	-	-	10	0.007	0.73
Copestylum flavipenne**	Aq.Sap	3	-	16	5	-	15	1	-	21	-	-	-	-	-	1	-	1	1	64	0.046	4.64
Copestylum fulvicorne*	Aq.Sap	-	-	-	-	-	1	-	-	-	-	-	-	-	-	-	-	-	-	1	0.001	0.07
Copestylum guianicum**	Aq.Sap	-	-	5	-	-	8	1	-	9	-	-	-	-	-	2	-	-	-	25	0.018	1.81
Copestylum hirtipes*	Aq.Sap	-	-	2	-	-	-	-	-	-	-	-	2	-	-	-	-	-	-	4	0.003	0.29
Copestylum mocanum*	Aq.Sap	8	-	8	8	-	65	-	-	3	-	-	-	3	-	2	-	-	-	97	0.070	7.03
Copestylum mus**	Aq.Sap	-	-	1	-	-	-	-	-	-	-	-	-	-	-	-	-	-	-	1	0.001	0.07
Copestylum musicanum*	Aq.Sap	25	-	28	21	-	225	-	-	3	4	-	4	6	-	15	-	-	-	331	0.240	24.00
Copestylum roraima*	Aq.Sap	-	-	1	1	-	3	-	-	-	-	-	1	-	-	-	-	-	-	6	0.004	0.44
Copestylum salti**	Aq.Sap	-	-	-	-	-	-	-	-	-	-	-	1	-	-	-	-	-	-	1	0.001	0.07
Copestylum sexmaculatum*	Aq.Sap	1	-	1	-	-	-	-	-	1	-	-	1	-	-	-	-	-	-	4	0.003	0.29
*Copestylum* sp. 1	Aq.Sap	-	-	1	8	-	13	-	-	1	-	-	-	-	-	2	-	2	-	27	0.020	1.96
*Copestylum* sp. 10	Aq.Sap	-	-	1	-	-	3	-	-	-	-	-	-	-	-	-	-	-	-	4	0.003	0.29
*Copestylum* sp. 11	Aq.Sap	1	-	-	-	-	-	-	-	-	-	-	-	-	-	-	-	-	1	2	0.001	0.15
*Copestylum* sp. 12	Aq.Sap	-	-	-	-	-	1	-	-	-	-	-	-	-	-	-	-	-	-	1	0.001	0,07
*Copestylum* sp. 13	Aq.Sap	-	-	-	-	-	-	-	-	1	-	-	-	-	-	-	-	-	-	1	0.001	0.07
*Copestylum* sp. 2	Aq.Sap	-	-	2	7	-	8	-	-	1	-	-	2	-	-	-	-	-	-	20	0.015	1.45
*Copestylum* sp. 3	Aq.Sap	-	-	-	-	-	-	-	-	1	-	-	-	-	-	-	-	1	-	2	0.001	0.15
*Copestylum* sp. 4	Aq.Sap	-	-	-	-	-	1	-	-	-	-	-	1	1	-	1	-	-	-	4	0.003	0.29
*Copestylum* sp. 5	Aq.Sap	1	-	-	-	-	-	-	-	-	-	-	-	-	-	-	-	-	-	1	0.001	0.07
*Copestylum* sp. 6	Aq.Sap	-	-	-	1	-	16	-	-	-	-	-	1	-	-	-	-	-	-	18	0.013	1.31
*Copestylum* sp. 7	Aq.Sap	-	-	-	-	-	-	-	-	-	-	-	1	-	-	-	-	-	-	1	0.001	0.07
*Copestylum* sp. 8	Aq.Sap	-	-	2	1	-	16	-	-	3	-	-	1	-	-	-	-	-	-	23	0.017	1.67
*Copestylum* sp. 9	Aq.Sap	-	-	-	-	-	2	-	-	-	-	-	-	-	-	-	-	-	-	2	0.001	0.15
*Copestylum spinithorax*	Aq.Sap	-	-	1	-	-	1	-	-	2	-	-	-	-	-	-	-	-	-	4	0.003	0.29
*Copestylum tenorium*	Aq.Sap	1	-	1	1	-	11	-	-	1	-	-	2	-	-	-	-	-	-	17	0.012	1.23
Copestylum tigrinum*	Aq.Sap	7	-	12	9	1	29	1	-	9	-	-	2	-	-	1	-	2	1	74	0.054	5.37
Copestylum trituberculatum**	Aq.Sap	1	-	4	3	-	6	-	-	4	-	-	-	-	-	1	-	-	-	19	0.014	1.38
Copestylum trivittatum**	Aq.Sap	-	-	1	-	-	-	-	-	1	-	-	1	-	-	-	-	1	-	4	0.003	0.29
*Copestylum tympanitis*	Aq.Sap	-	-	5	4	-	4	-	-	14	-	-	1	-	-	-	-	2	4	34	0.025	2.47
*Copestylum vagum*	Aq.Sap	21	-	25	21	-	179	1	-	15	1	-	5	11	-	14	-	-	5	298	0.216	21.61
*Copestylum virescens*	Aq.Sap	-	-	-	-	-	-	-	-	-	-	-	-	1	-	-	-	-	-	1	0.001	0.07
*Copestylum viride*	Aq.Sap	-	-	-	-	-	-	-	-	1	-	-	-	-	-	-	-	-	-	1	0.001	0.07
Copestylum willistoni**	Aq.Sap	-	-	-	-	-	1	-	-	-	-	-	-	-	-	-	-	-	-	1	0.001	0.07
Ornidia major*	Ter.Sap	3	-	13	1	-	-	1	-	2	-	-	1	1	-	-	-	-	-	22	0.016	1.60
*Ornidia obesa*	Ter.Sap	1	-	2	-	-	-	1	-	4	-	-	1	-	-	-	-	-	-	9	0.007	0.65
Palpada agrorum*	Aq.Sap	-	-	-	-	-	1	-	-	-	-	-	-	-	-	-	-	-	-	1	0.001	0.07
Palpada fasciata**	Aq.Sap	-	-	-	-	-	1	-	-	-	-	-	-	-	-	-	-	-	-	1	0.001	0.07
Palpada inversa**	Aq.Sap	-	-	-	-	-	1	-	-	-	-	-	-	-	-	-	-	-	-	1	0.001	0.07
Palpada scutellaris*	Aq.Sap	-	-	-	-	-	-	1	-	-	-	-	-	-	-	-	-	-	-	1	0.001	0.07
*Palpada vinetorum*	Aq.Sap	-	-	-	-	-	-	-	-	1	-	-	-	-	-	-	-	-	1	2	0.001	0.15
*Quichuana angustiventris*	Aq.Sap	-	-	-	-	-	10	-	-	-	-	-	-	-	-	-	-	-	-	10	0.007	0.73
Quichuana picadoi*	Aq.Sap	-	-	-	-	-	-	-	-	-	-	-	-	-	-	-	-	1	-	1	0.001	0.07
Sterphus plagiatus*	Aq.Xil	-	-	1	-	-	-	-	-	1	-	-	-	-	-	-	-	-	-	2	0.001	0.15
**Syrphinae**																						
Argentinomyia longicornis*	Ter.Zoo	-	-	-	-	-	2	-	-	-	-	-	-	-	-	-	-	-	-	2	0.001	0.15
*Dioprosopa clavata*	Ter.Zoo	-	-	-	-	-	-	1	-	-	-	-	-	-	-	-	-	-	-	1	0.001	0.07
Hybobathus flavipennis*	Ter.Zoo	1	-	-	-	-	1	1	1	10	-	-	-	-	-	-	-	-	1	15	0.011	1.09
Hybobathus lineatus*	Ter.Zoo	-	-	-	-	-	1	-	-	1	-	-	-	-	-	-	-	-	-	2	0.001	0.15
Hybobathus lividus*	Ter.Zoo	1	-	-	1	-	-	-	-	-	-	-	-	-	-	-	-	-	-	2	0.001	0.15
Hybobathus norina*	Ter.Zoo	-	-	-	-	-	-	-	-	2	-	-	-	-	-	-	-	-	1	3	0.002	0.22
Hybobathus persimilis*	Ter.Zoo	-	-	-	-	-	-	-	-	1	-	-	-	-	-	-	-	-	-	1	0.001	0.07
Ocyptamus dimidiatus*	Ter.Zoo	-	-	-	-	-	-	1	-	1	-	-	-	-	-	-	-	-	-	2	0.001	0.15
Ocyptamus gastrostactus*	Ter.Zoo	-	2	-	-	-	-	1	-	-	-	-	-	-	-	-	-	-	-	3	0.002	0.22
Pelecinobaccha adspersa*	Ter.Zoo	-	1	1	-	-	-	2	-	1	-	-	-	-	-	-	-	-	-	5	0.004	0.36
Pelecinobaccha eruptova**	Ter.Zoo	-	-	-	-	-	1	-	-	1	-	-	-	-	-	-	-	-	-	2	0.001	0.15
Relictanum crassum*	Ter.Zoo	-	-	-	-	-	1	-	-	-	-	-	-	-	-	-	-	-	-	1	0.001	0.07
Relictanum nero*	Ter.Zoo	-	-	-	-	-	-	-	-	1	-	-	-	-	-	-	-	-	-	1	0.001	0.07
Salpingogaster nigra*	Ter.Zoo	4	-	1	4	-	1	3	-	32	-	-	-	-	-	5	2	-	2	54	0.039	3.92
Toxomerus floralis*	Ter.Zoo	-	-	-	-	-	3	-	-	-	-	-	-	-	-	-	-	-	-	3	0.002	0.22
Toxomerus productus**	Ter.Zoo	-	1	-	-	-	-	-	-	-	-	-	-	-	-	-	-	-	-	1	0.001	0.07
Toxomerus pulchellus*	Ter.Zoo	-	-	-	-	-	-	1	-	2	-	-	-	-	-	-	-	-	-	3	0.002	0.22
Toxomerus virgulatus*	Ter.Zoo	1	-	-	-	-	-	-	-	1	-	-	-	-	-	-	-	-	1	3	0.002	0.22
**Abundance**		**84**	**4**	**144**	**116**	**1**	**695**	**22**	**1**	**173**	**6**	**0**	**31**	**24**	**0**	**47**	**2**	**11**	**18**	**1,379**	**1**	**100**
**Total number of genera**		**4**	**3**	**5**	**4**	**1**	**8**	**9**	**1**	**10**	**1**	**0**	**2**	**2**	**0**	**2**	**1**	**2**	**5**	**13**		
**Total number of species**		**19**	**3**	**31**	**20**	**1**	**41**	**17**	**1**	**38**	**3**	**0**	**20**	**7**	**0**	**13**	**1**	**8**	**10**	**72**		

A.A. = Absolute abundance, R.A. = Relative abundance, R.F.% = Relative frequency. Larval trophic categories: Aq.Sap = Saprophagous aquatic, Aq.Xil = Xilosaprophagous aquatic, Ter.Sap = Saprophagous terrestrial, Ter.Zoo = Zoophagous terrestrial. *Extension of distribution for the Colombian Amazon region, **New record for Colombia

*Copestylum* Macquart was the most abundant and diverse genus in the DSF (*n* = 848; 61.5% of all specimens, distributed in 31 species), followed by *Quichuana* Knab (*n* = 11, 2 species) and *Salpingogaster* Schiner (*n* = 10, 1 species). In the FE and AFS, *Copestylum* was also the best-represented genus with 33 and 25 species, respectively (*n* = 234 and 143; 16.9% and 10.4% of all specimens). Species were considered dominant (with ≥ 20 individuals), finding two abundant species in FE and AFS—HIR, and eight in DSF—HIR, meanwhile in LIR the only habitat that presented abundant species was DSF with two. In terms of exclusive species, the best-represented habitats were DSF (*n* = 13) and AFS (*n* = 12) during HIR, while the lowest number of exclusive species was found in DSF during LIR (Table 2).

**Table 2:** Diversity indicators for community assemblages of adult flower flies (Diptera: Syrphidae) and estimador sample coverage (SC) per habitat of La Avispa Nature and Ecotourism Reserve, Caquetá, Colombia, during two rainy seasons (HIR = High-intensity rainfall and LIR = Low-intensity rainfall) from February to May of 2022

Indicator^*a*^			Habitat			
	Forest Edge		Dense Secondary Forest		Agroforestry System	
	HIR	LIR	HIR	LIR	HIR	LIR
Total abundance (TA)	232	37	812	71	196	31
Number of genera	8	2	10	3	11	6
Number of exclusive genera	0	0	1	0	1	0
Number of species (S)	38	21	43	16	41	15
Number of exclusive species	5	3	13	1	12	4
Number of dominant species	2	0	8	2	0	2
Estimator of the sample coverage	0.92	0.71	0.98	0.87	0.90	0.71
S interpolated	38	20	43	16	41	15
Interpolation completeness	1	0.95	1	1	1	1
S extrapolated	48	32	53	22	57	22
Extrapolation completeness	0.79	0.65	0.81	0.72	0.71	0.68

^*a*^TA = cumulative number of specimens of all species and morphospecies captured, dominant species with ≥ 20 individuals

The absolute abundance of flower flies was almost three times higher in the DSF (*n* = 883; 64% of all specimens) than in FE (*n* = 269; 19.5%), and almost four times higher compared with the AFS (*n* = 227; 16.5%) (Table 2). The AFS obtained the highest number of genera (*n* = 12; 92% of the genera recorded). The DSF and AFS exhibited the same richness (44 species), representing 12.2% of the Colombian fauna. The FE presented the lowest diversity (43 species); however, it was not so dissimilar from that exhibited by the other two habitats evaluated. Discriminating abundance by the rainy season, the DSF has the highest abundance in both rainy seasons (65.5% and 51.1% of all specimens for HIR and LIR, respectively). The lowest abundance was found in the AFS (*n* = 196; 15.8% and *n* = 31; 22.3% of all specimens for HIR and LIR, respectively). In the three habitats, the flower fly diversity was higher in HIR than in LIR. In HIR, the number of dominant species (with ≥ 20 individuals) was higher in DSF (*n* = 8) than in FE and AFS, both with 2 dominant species, while in LIR only DSF presented dominant species (*n* = 2) (Table 2).

**Fig. 2:**
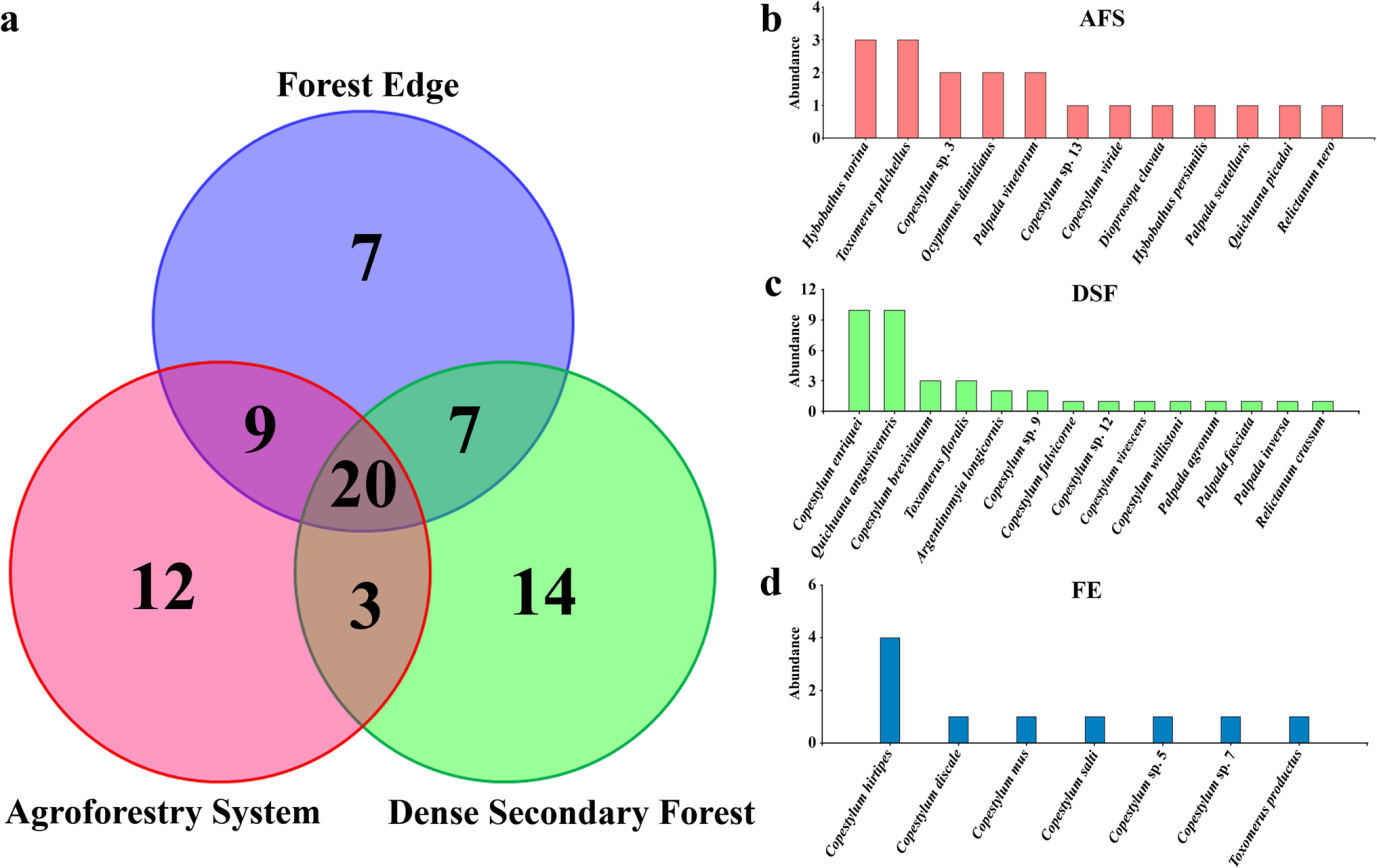
Faunal composition of adult flower flies (Diptera: Syrphidae) in the three habitats (AFS = Agroforestry System, DSF = Dense Secondary Forest, and FE = Forest Edge) in La Avispa Nature and Ecotourism Reserve, Caquetá, Colombia from February to May of 2022. **a**. Venn diagram shows the number of exclusive and shared species for each habitat. Abundance of exclusive species for each habitat: **b**. AFS, **c**. DSF, **d**. FE. The size of the bar is proportional to the abundance (total number of captured adults) of each species

According to the sample coverage estimator (SC), sampling effort during HIR in all habitats reached 90% or more, with DSF having the best representativeness (SC = 0.98) (Table 2). All values obtained for the estimator of sample coverage During LIR, the SC was equal 71% only in FE and AFS, while those for extrapolation completeness during HIR, averaged 65% and 68% in LIR, respectively. On the other hand, during the HIR, the highest expected richness was in the AFS (S extrapolated = 57), while in LIR it was FE (32). The only habitat that did not obtain 100% of interpolation completeness was FE during the LIR. The highest values of extrapolation completeness were estimated for the DSF during both rainy season. The sampling representativeness in the three habitats (covering the two rainy seasons) exceeded 92%, with the DSF being the most complete (98%), followed by FE (92.9%) and AFS (92.5%). Mean completeness during HIR was 93.3%, while in LIR it was 76.3% (Supplementary Material: Fig. S2). No exclusive genus was found in FE during both rainy season, nor in DSF and AFS in LIR. One exclusive genus was found only in the DSF (*Argentinomyia* Lynch-Arribálzaga) and AFS (*Dioprosopa* Hull), which occurred during HIR. Regarding exclusive species, 12 species were exclusively collected in the AFS (Figs. 2a and b), 14 species were only recorded in the DSF, with three species recorded for the first time in Colombia (Figs. 2a and c), and FE has seven exclusive species, with four species are also new records for the country (Figs. 2a and d).

**Fig. 3:**
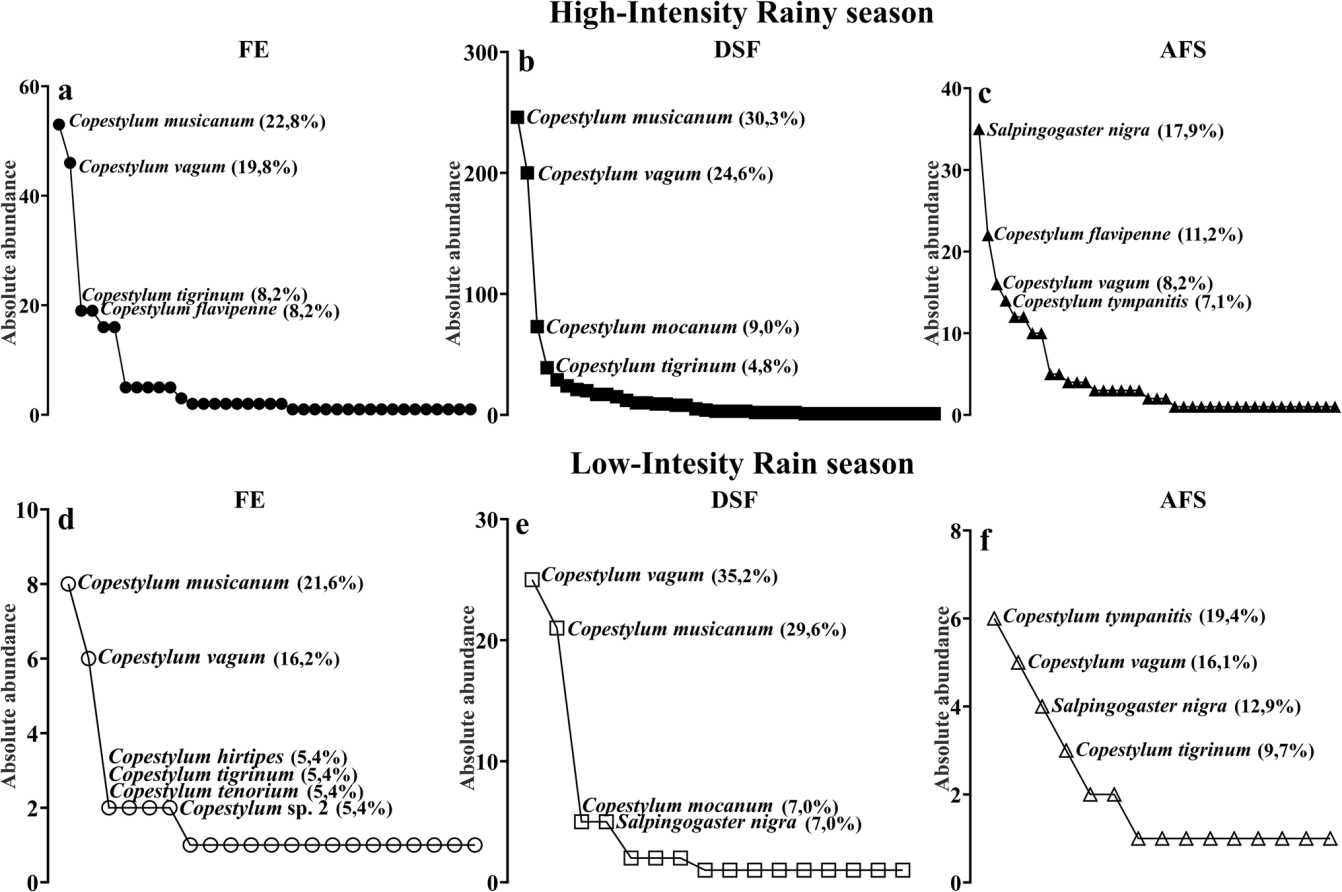
Contracts among the community structures of adult flower flies (Diptera: Syrphidae) in the three habitats (FE = Forest Edge, DSF = Dense Secondary Forest, and AFS = Agroforest System) in La Avispa Nature and Ecotourism Reserve, Caquetá, Colombia, based on rank-abundance curves, during the two rainy seasons: high-intensity rainfall (**a**, **b** and **c**) and low-intensity rainfall (**d**, **e** and **f**)

Regarding the structure of the flower fly community, *Copestylum musicanum* (Curran) and *Copestylum vagum* (Wiedemann) had a co-dominance pattern, with alternation between habitats and rainy seasons, followed by *C*. *tigrinum* Ricarte and Hancock (Fig. 3). Examining the distribution of flower fly abundances throughout habitats and rainy seasons, absolute abundances tend to be uniform and the dominant species were the same for FE and DSF in the HIR (Fig. 3a-b), while the AFS was dominated by *Salpingogaster nigra* Schiner (Fig. 3c). During LIR, the absolute abundances of *C*. *musicanum* and *C*. *vagum* in DSF were significantly different from the other species, while the representativeness of *C*. *vagum* in the other two habitats was similar (Fig. 3d-f). *Copestylum musicanum* dominated in FE (Fig. 3d), whereas *C*. *vagum* dominated in DSF (Fig. 3e) and *C*. *tympanitis* (Fabricius) in the AFS during LIR (Fig. 3f).

Comparing the values of the three Hill numbers for each habitat in the two rainy seasons sampled, the highest richness of flower flies was found for DSF during the HIR (q0 = 43.0), followed by AFS (q0 = 41.0) and FE (q0 = 38.0). Richness for the rainy season was not significantly different between habitats (*p-value* > 0.05). During the HIR, the values q1 and q2 for AFS (21.2 and 13.5, respectively) were higher than those obtained for FE (q1 = 14.5; q2 = 8.4) and DSF (q1 = 11.1; q2 = 5.9). In the LIR season, the highest richness was obtained at the FE (q0 = 21.0), which differed from the DSF (q0 = 16.0) and AFS (q0 = 15.0). Considering the q1 and q2 numbers, the Syrphidae fauna in the FE presented values (15.1 and 10.4, respectively) that significantly differed from the values for DSF (6.9 and 4.4, respectively) during the LIR, however, not significant differences were found among the q1 and q2 values for AFS (11.6 and 9.3, respectively) (Supplementary Material Fig. S2).

**Fig. 4:**
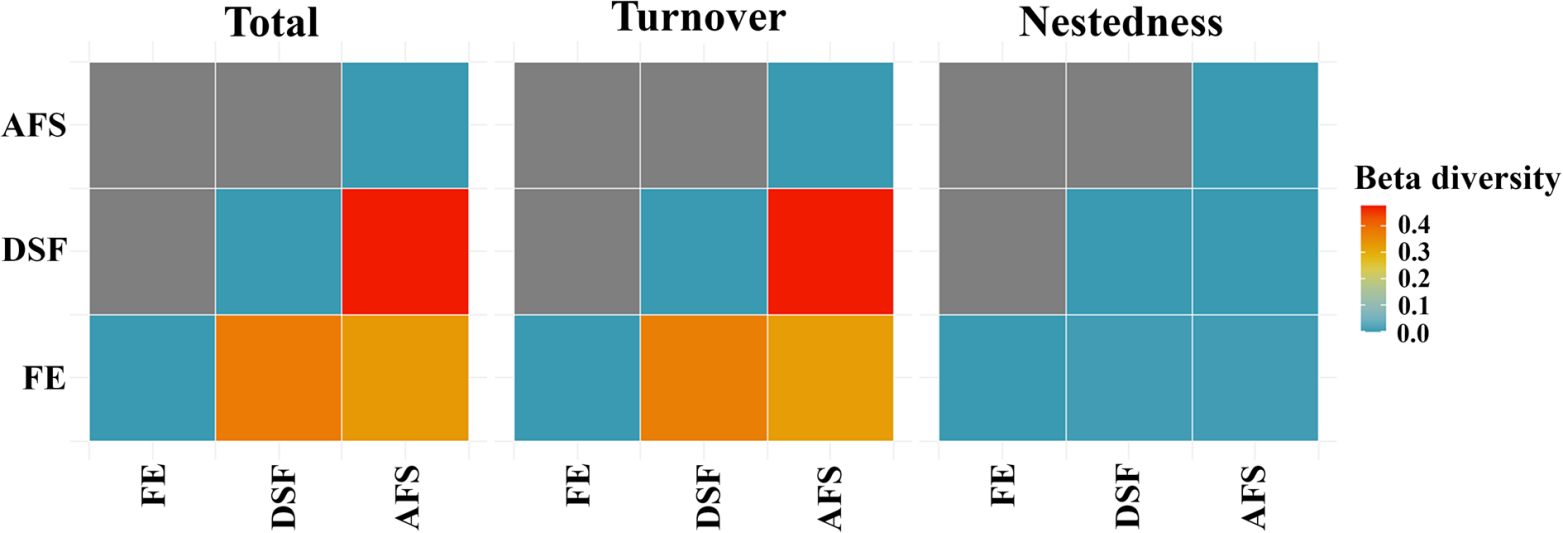
Beta diversity of adult flower flies (Diptera: Syrphidae) (turnover = “species replacements” and nestedness = “species lost”, which usually are not replaced) and pairwise comparison of habitats (AFS = Agroforestry System, DSF = Dense Secondary Forest, and FE = Forest Edge) in La Avispa Nature and Ecotourism Reserve, Caquetá, Colombia from February to May of 2022. Beta diversity (*β*) = 0.0 (comparing an assemblage to itself)

**Fig. 5:**
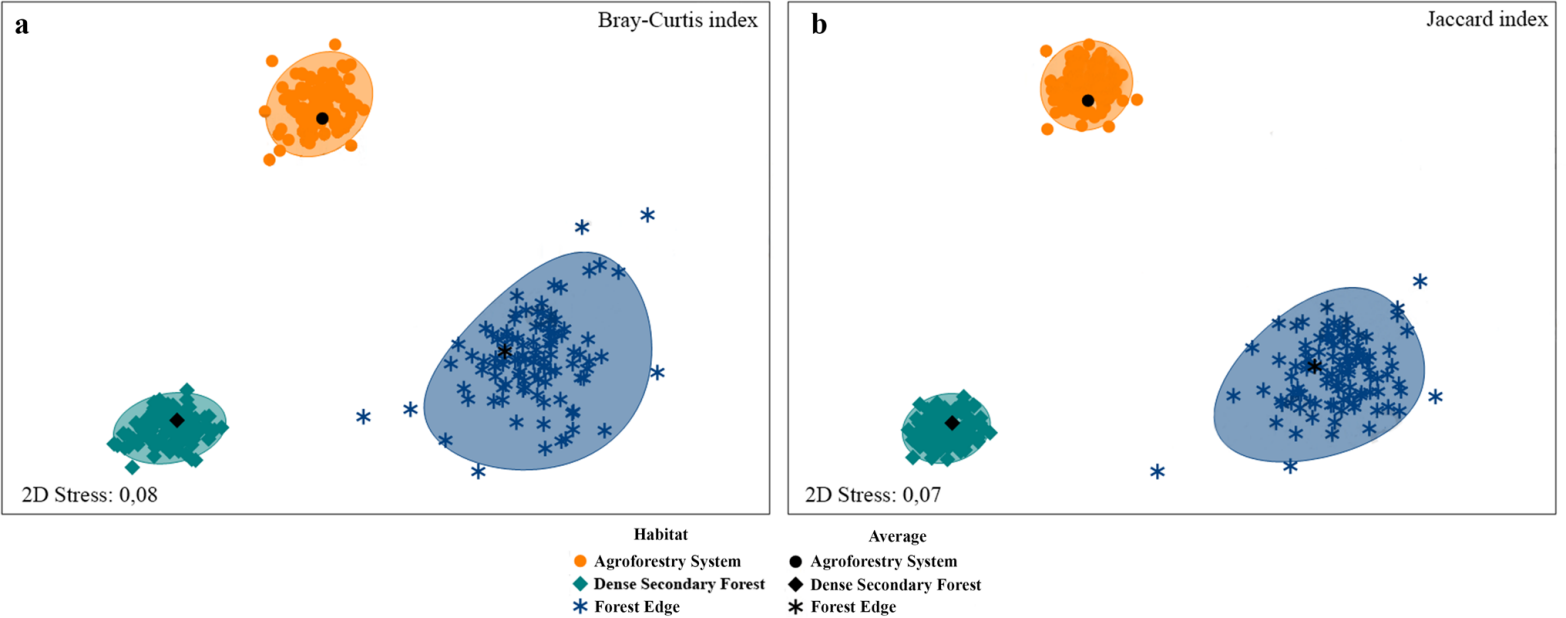
Similarity of communities of adult flower flies (Diptera: Syrphidae) between the three habitats (Agroforestry System, Dense Secondary Forest and Forest Edge) in La Avispa Nature and Ecotourism Reserve, Caquetá, Colombia from February to May of 2022, based on non-metric multidimensional scaling (NMDS) ordination for habitats defined by two indices: **a**. Bray–Curtis (quantitative index), **b**. Jaccard (qualitative index) for the abundance (total number of captured adults) of flower fly species. Similarity differences were assessed using permutational multivariate analysis of variance (PERMANOVA) with 9,999 permutations of residuals

Beta diversity of flower flies in the reserve was dominated by species turnover between and within habitats (Fig. 4). A pairwise comparison of habitats yielded that beta diversity was 56.3%, dominated by species turnover rather than nestedness. The greatest dissimilarity between habitats was observed between DSF and AFS (0.64) (Supplementary Material: Table S2). Differences in flower fly species composition were detected for the habitats, using the similarity indices of Bray–Curtis (Stress = 0.08) (Fig. 5a) and Jaccard (Stress = 0.07) (Fig. 5b), which were compared with a PERMANOVA analysis, where Bray–Curtis (*Pseudo-F* = 0.78; df = 2) and Jaccard (*Pseudo-F* = 1.26; df = 2) ratified the dissimilarity between habitats.

### Phenology of the Activity Period of Flower Flies

**Fig. 6:**
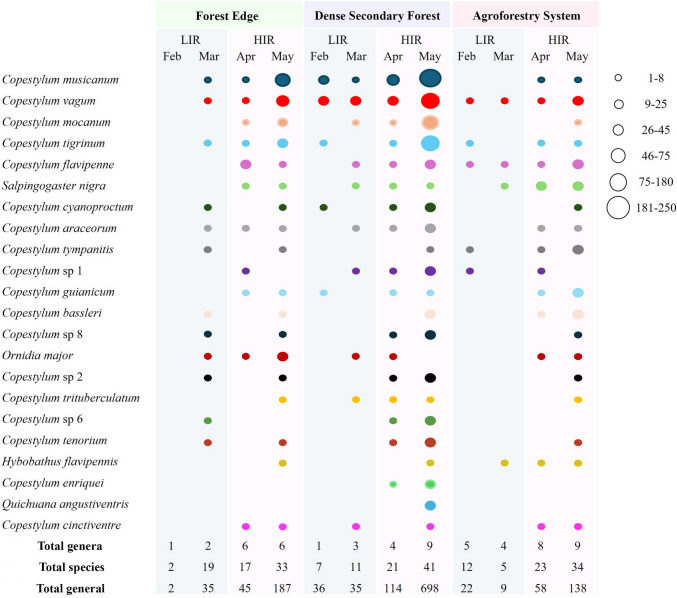
Phenology of the activity of flower fly species (Diptera: Syrphidae) occurring in the three habitats (Forest Edge, Dense Secondary Forest and Agroforestry System) in La Avispa Nature and Ecotourism Reserve, Caquetá, Colombia from February to May of 2022. The size of the circles is proportional to the abundance (total number of adults captured monthly) of flower fly species obtained in each habitat and rainy season (HIR = High-intensity rainfall and LIR = Low-intensity rainfall)

Of the 72 species (59 valid names and thirteen morphospecies) recorded in this study, 33 occurred in both rainy seasons (Table 1). In comparison, 35 species were recorded exclusively during HIR and four (*Copestylum* sp. 7, *C*. *virescens* (Williston), *C*. *salti* (Curran) and *Quichuana picadoi* Knab) during LIR. Only the 22 species had an abundance ≥ 10 individuals and they were included for phenology analysis (Fig. 6). It is noteworthy that none of these species were recorded during February 2022 (LIR) in the FE. In terms of occurrence, 21 species were identified for the first time this month, mainly collected in the AFS (*n* = 22, 12 species). *Copestylum musicacum*, *C*. *trigrinum* and *C*. *vagum* occurred in all habitats in both rainy seasons. On the other hand, *C*. *cinctiventre* (Curran) was also present in the three habitats, but only during HIR.

### Effectiveness of Baits in Capturing Flower Flies and the Functional Groups of Their Larvae

**Fig. 7:**
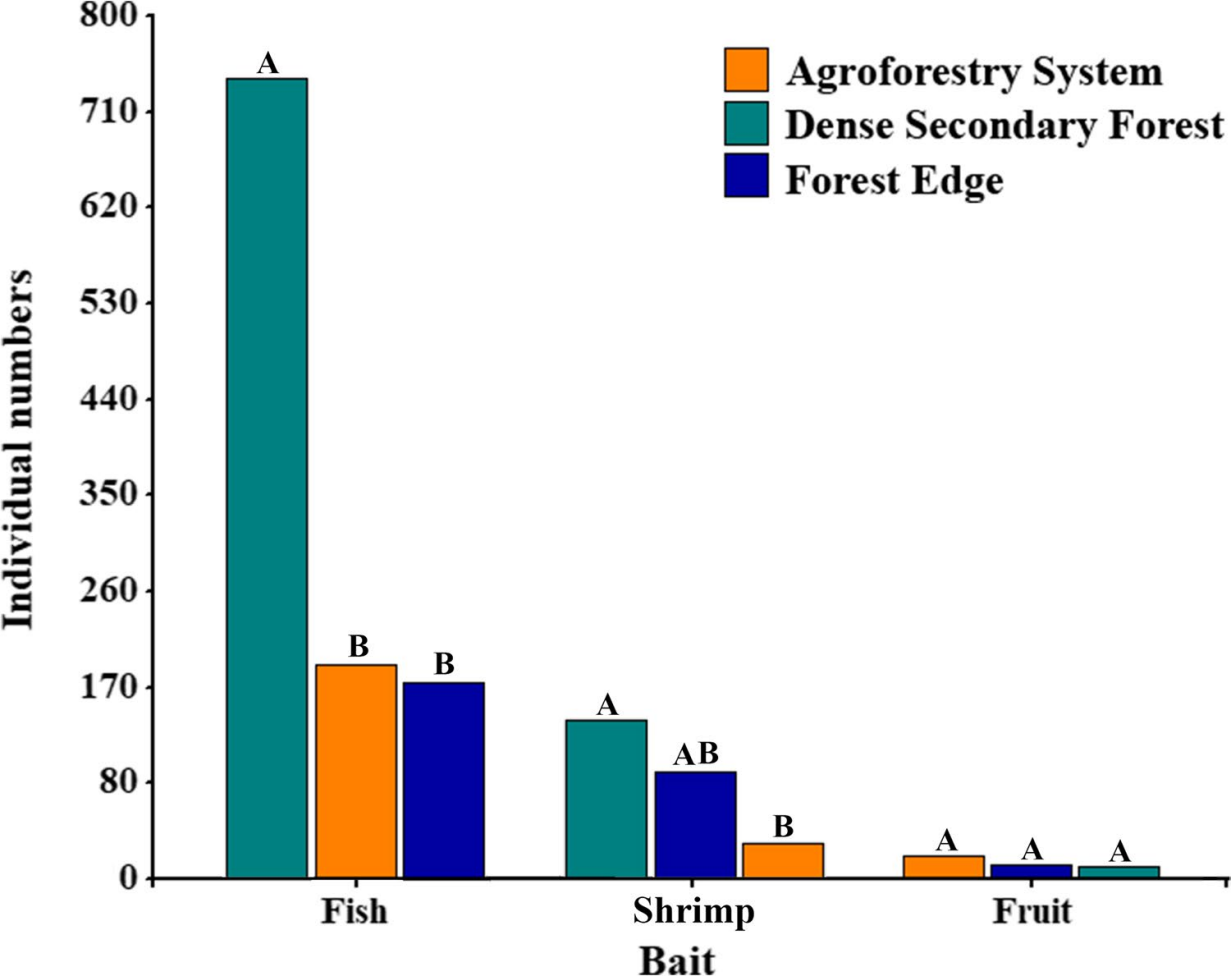
Effectiveness of the baits (Fish = Decomposing fish, Shrimp = decomposing shrimp and Fruit = fermented banana-pawpaw fruits) used in the Van Someren-Rydon traps to collect adult flower flies (Diptera: Syrphidae) in the three habitats (Agroforestry System, Dense Secondary Forest and Forest Edge) of La Avispa Nature and Ecotourism Reserve, Caquetá, Colombia from February to May of 2022. The size of the bar is proportional to the number of individuals collected (three replications per trap for each habitat)

**Fig. 8:**
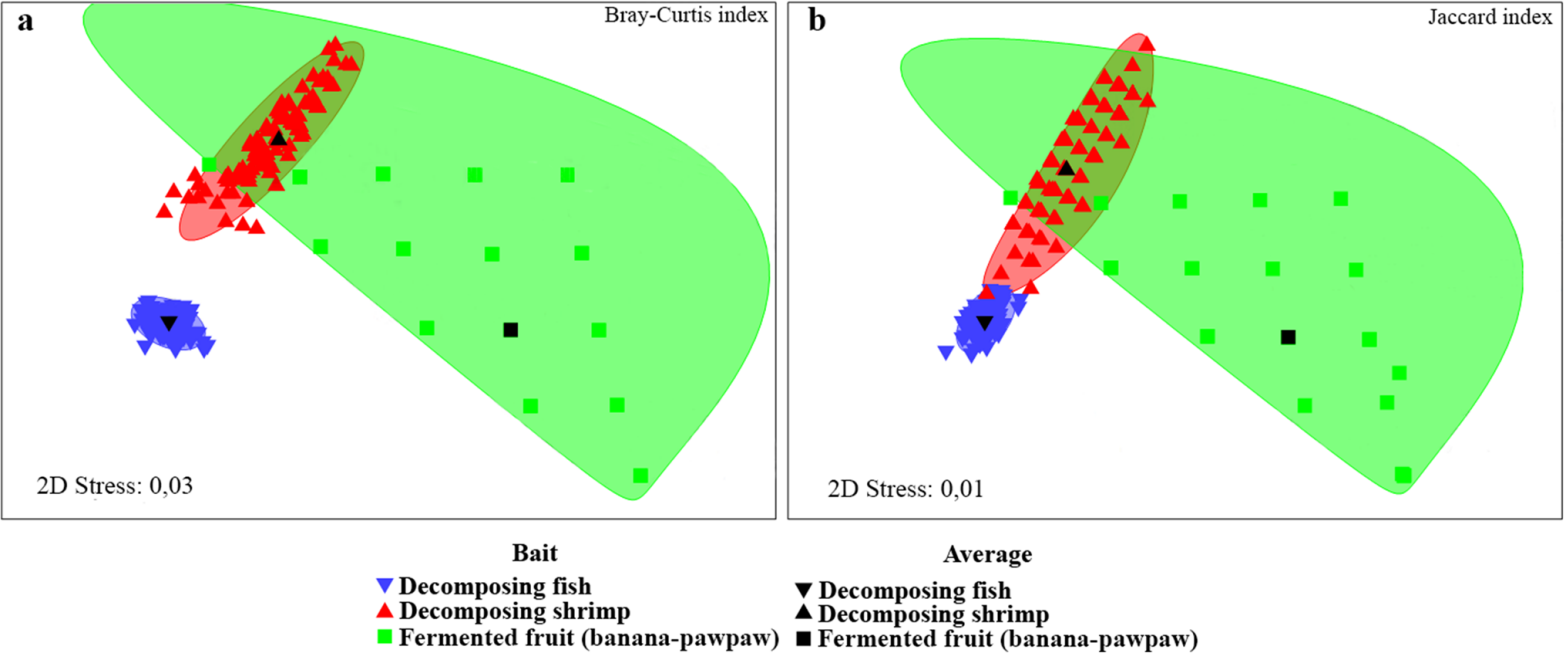
Non-metric multidimensional scaling (NMDS) ordination of baits (Decomposing fish, decomposing shrimp and fermented banana-pawpaw fruits) used in the Van Someren-Rydon traps to collect adult flower flies (Diptera: Syrphidae) in the three habitats (Agroforestry System, Dense Secondary Forest and Forest Edge) of La Avispa Nature and Ecotourism Reserve, Caquetá, Colombia from February to May of 2022. NMDS ordination was defined by two indices of similarity: a. Bray–Curtis and b. Jaccard (qualitative index) for richness of flower fly species. Similarity differences were assessed using permutational multivariate analysis of variance (PERMANOVA) with 9,999 permutations of residuals

The Kruskal–Wallis test (*p*-value = 0.0003) showed that the control (fruit) was significantly different from the meat-based baits (fish and shrimp) (Supplementary Material: Table S3). Demonstrating that the most effective bait for was decomposing fish, collecting a total of 1,108 specimens (80% of the total sampled) distributed across 64 species (89% of the total species identified). Greater effectiveness occurred in DSF, where it captured 41 species (57%) and 722 specimens (52%) (Fig. 7). The second most effective bait was decomposing shrimp, collecting 37 species (51%) and 254 individuals (18%), showing greater effectiveness in DSF, with 23 species (32%) and 140 specimens (10%). Fermented fruit was less effective (*n* = 17; 1%), reaching a maximum representativeness of 12 specimens (5%), belonging to 9 species (13%) in the AFS. Adult flower flies showed a great preference for decomposing fish during the two rainy seasons, obtaining preference values above 86%, being HIR, the rainy season with the highest observed percentage (89.7%; 61 of 68 species collected during the season), while during LIR reached 86.5% (32 of 37 species collected), becoming the preferred bait for flower flies (Table 1; Fig. 7). In the preference analysis carried out by rainy seasons, only species with abundances ≥ 10 individuals were taken into account, where it was observed that in most cases showed preferences by decomposing fish, in both rainy seasons. However, in some species such as *Copestylum mocanum*, the bait preference changed with the rainy season, reaching up 82.6% preference for fish and 17.4% for decomposing shrimp during HIR, to 40% and 60%, during LIR, respectively. On the other hand, *C*. *flavipenne*, *C*. sp. 1, *C*. *tigrinum* and *C*. *tympanitis* in HIR showed preferences for decomposing fish and shrimp, while in LIR, it was decomposing fish and fermented fruit. Additionally, it was observed that the species distribution concerning bait preferences suggests that decomposing shrimp and fermented fruit overlap, according to the Bray–Curtis (Stress = 0.03) (Fig. 8a) and Jaccard (Stress = 0.01) indices (Fig. 8b). However, PERMANOVA analysis employing the Bray–Curtis (*Pseudo-F* = 0.9; df = 2) and Jaccard (*Pseudo-F* = 0.85; df = 2) indices revealed that there are significant differences between baits and species distribution.

**Fig. 9:**
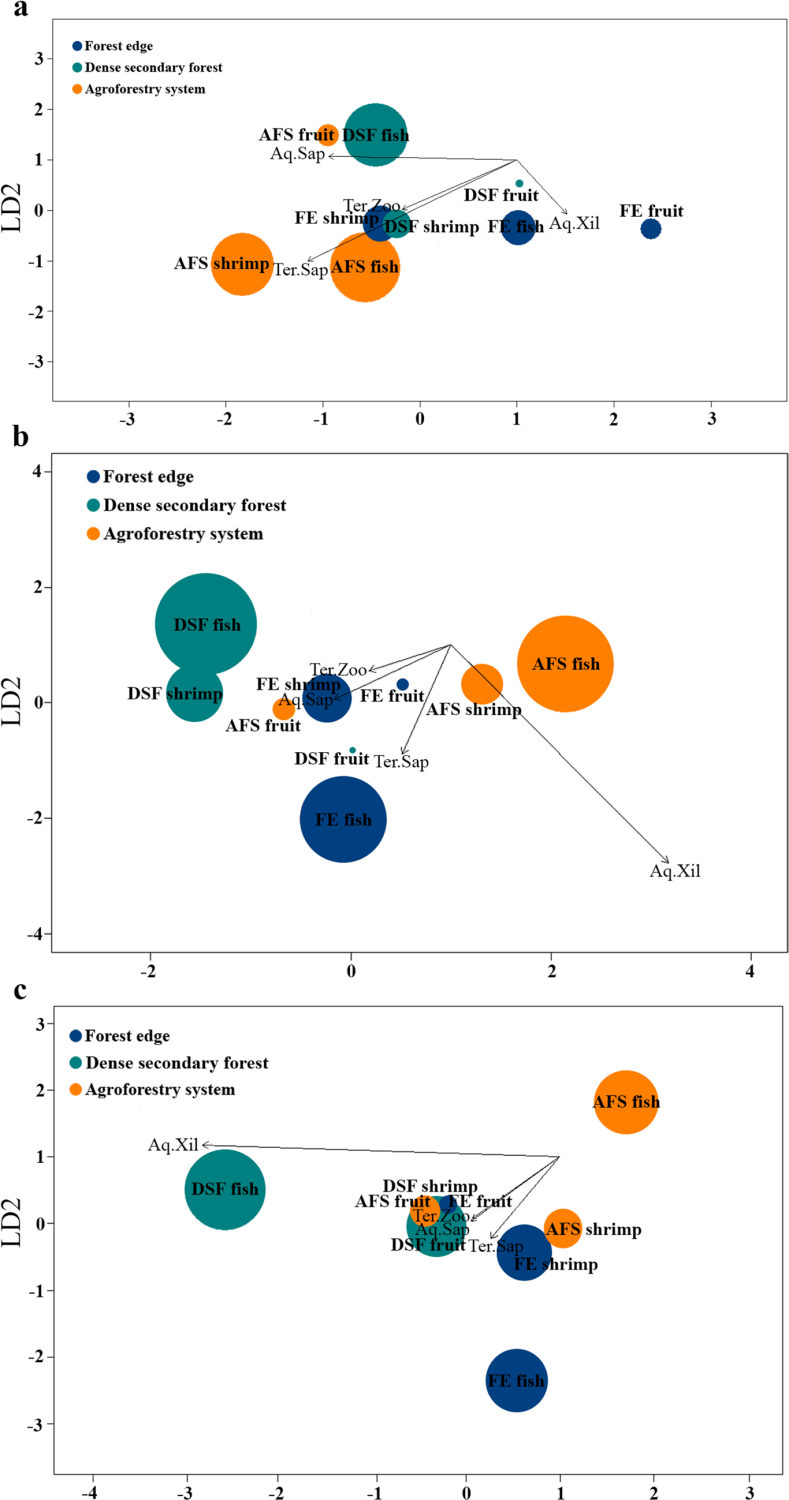
Linear discriminant analyses for bait preference by adult flower flies (Diptera: Syrphidae), their larval trophic category and habitats of La Avispa Nature and Ecotourism Reserve, Caquetá, Colombia from February to May of 2022. The size of the dots in each subpanel is proportional to the number of genera (**a**), species (**b**) and individuals (adults) (**c**) collected in the three habitats (Agroforestry System, Dense Secondary Forest and Forest Edge). Baits: Decomposing fish, decomposing shrimp and fermented banana-pawpaw fruits used in the Van Someren-Rydon traps to collect adults. Larval trophic category: Aq.Sap = Saprophagous aquatic, Aq.Xil = Xilosaprophagous aquatic, Ter.Sap = Saprophagous terrestrial, and Ter.Zoo = Zoophagous terrestrial. Black lines represent the magnitude (length) and sign (direction) of the correlation of each larval trophic category with each discriminant axis. The analyses considered the abundance and richness of exclusive and shared species between each habitat

DSF and AFS harbor the highest number of genera and species richness, as well as the highest diversity of the larval trophic category, compared to the FE (Table 1; Fig. 9). In terms of richness, the larval trophic category with the highest diversity and abundance was aquatic saprophagous (2 genera, 46 species, *n* = 1.236, 89.6%) (Fig. 9a), followed by terrestrial zoophagous (8 genera, 18 species, *n* = 104, 7.5%) and terrestrial saprophagous (2 genera, 7 species, *n* = 37, 2.7%) (Fig. 9b-c). Aquatic xilosaprophagous (1 genus, 1 species, *n* = 2, 0.1%) had the lowest diversity, exclusively recorded in the FE and AFS (Fig. 9c).

## Discussion

### Abundance, Diversity and Phenology of Flower Flies

Knowledge about the diversity of Syrphidae in the Colombian Amazon region is still incipient, with only one study recording the expansion of the geographic distribution of *Cepa apeca* Thompson (Diptera, Syrphidae, Eristalinae), which is a species considered rare (Parada-Marín et al. 2021), as well as the description of *Alipumilio aureus* Parada-Marin, Mengual and Ramos-Pastrana, 2024 and *C. enriquei* Montoya, Parada-Marín and Ramos-Pastrana, 2022, endemic species to the Amazonian region (Montoya et al. 2012, 2021; Parada-Marín et al. 2021; Parada-Marin et al. 2024). *C. enriquei* was exclusively collected together with *Quichuana angustiventris* (Macquart) in the DSF during HIR. Our study records 59 valid species and thirteen morphospecies for La Avispa Nature and Ecotourism Reserve, corresponding to 20% of the Colombian Syrphidae fauna (Montoya et al. 2012, 2021, 2022; Montoya 2016; Parada-Marín et al. 2021; Montoya and Wolff 2023), including, 16 species recorded for the first time in the country, increasing the Syrphidae fauna of Colombia to 384 species, including species that extend their distribution range to the Amazon region as well as other potentially new to science.

The three habitats of the reserve were dominated by *Copestylum* species (Aquatic saprophagous as larva), a New World endemic genus that harbor the second most diverse genus of the Syrphidae, with more than 400 described species and many more undescribed (Thompson 1981; Rotheray et al. 2007; Ricarte et al. 2015; Montoya et al. 2022), of which 71 have been recorded in Colombia (Restrepo-Ortiz and Carrejo 2009; Montoya 2016; Montoya et al. 2021, 2022). In our study, 12 species of this taxon are recorded for the first time in the country, highlighting the strategic importance of the studied reserve for biodiversity conservation. The AFS at HIR was the only habitat dominated by a distinct species of *Copestylum* (*S. nigra*; *n* = 35).

*Salpingogaster nigra* (Terrestrial zoophagous as larva) reached its highest abundance (*n* = 39) in the AFS during HIR, which could be because the species is considered the natural enemy and main biological controller of the spittlebug, *Aeneolamia varia* (Fabricius) (Hemiptera: Cercopidae), which can prey up to 17–40 nymphs during its larval stage and reaching up three generations per life cycle (Guppy 1913; Sotelo and Cardona 2001; Bustillo 2011; Espitia et al. 2022) in sugarcane crop (Páez et al. 1985; Lastra et al. 2007), which were the dominant botanical species in the AFS. Thus, the prevalence of *S. nigra* during HIR could be related to the higher availability of spittlebugs as its prey in this season. According to Veríssimo et al. (2018), this syrphid tends to decrease considerably during the dry season. *Dioprosopa clavata* (Fabricius) was exclusive to AFS during HIR. This species is widely distributed in tropical and subtropical areas of America (Kassebeer 2000; Lillo et al. 2021), being listed as a natural enemy of numerous aphid species of agricultural importance, considered also as biological control of spittlebug of the genus *Aeneolamia* Fennah (Hemiptera: Cercopidae), one of the main pest in sugarcane crops (Rojo et al. 2003; Arcaya et al. 2018; Lillo et al. 2021; Ferrer and Salas 2024).

In the interpolation and extrapolation curves, the highest completeness, concerning the sample coverage estimator was obtained by the DSF (98%), possibly due to the constant rainfall during HIR, which causes an increase in water reservoirs, especially phytotelmata, providing decomposing organic matter and creating optimal conditions for the development of saprophagous larvae of Eristalinae (*Copestylum*, *Palpada* Macquart and *Quichuana*) (Howarth and Edmunds 2000; Rotheray et al. 2007), subfamily that presented the highest richness during the rainy season (*n* = 1,242, 47 species). The FE obtained a completeness of 92.9%, the same value obtained by the AFS, attributable to the prevalence of some species that prefer opened areas, with high light incidence (Souza et al. 2014). In Montoya et al. (2021), grassland habitats, characterized by large open areas, obtained the lowest number of genera (23) and species (59), therefore, in our study, this type of habitat obtained the lowest completeness concerning the other sampled habitats. Despite this, grassland was the second most abundant habitat in flower flies (*n* = 571), representing 20% of the collected material. However, the authors state that anthropization, plant homogeneity and extreme climatic conditions could impact diversity, with the prevalence of generalist and widely distributed species. The AFS obtained the same richness as the DSF (44 species), however, the lowest completeness (92.5%). Data that contrast with those obtained by Souza et al. (2014), where the Forest Edge was the most abundant and diverse habitat, with Syrphinae species being the best represented, suggesting the preference of species of this taxon for open areas.

Lower values SC during LIR and extrapolation completeness during HIR for AFS and FE may be due to increased temperature that could lead to a decrease in water reservoirs, which in turn, dries the decomposing organic material and limits the survival of populations of other key insects. This environmental condition is critical for the development of some species of Syrphinae (especially terrestrial zoophages), limiting the persistence of specialist functional groups (Gittings et al. 2006; Meyer et al. 2009; Montoya et al. 2021).

The Hill numbers found in our study suggested a greater richness during HIR, corroborating the result of Noriega et al. (2021), who indicated that high rainfall promotes more abundant and diverse insect assemblages, due to environmental conditions, which considerably impact plant heterogeneity in conserved habitats, contributing to stability and increasing diversity, meanwhile, the dry season and the plant homogeneity that characterizes disturbed habitats causes a decrease in insect biodiversity. Sommaggio (1999) reported that the diversity of Syrphidae is determined by the availability of food resources for their immature stages. In our study, we were able to identify the presence of four larval trophic categories, in which richness at genera and species level, as well as abundance was skewed, with terrestrial zoophagous having the highest genera number (*n* = 8), being the second most speciose group (*n* = 18). This group was equally diverse at Santa-Inés-Belmira and Sonsón Paramo complexes in Colombia, recording 22 genera and 84 species (Montoya et al. 2021). Likewise, Djellab et al. (2019) stated that seasonality has a considerable impact on the syrphid fly composition in pine forests (*Pinus halepensi*s Miller) of Algeria, North Africa, as it influences food resources and availability of developmental microhabitats, directly intervening in population dynamics. The high diversity obtained during HIR may be attributable to the increase in water reservoirs formed in phytotelmata, providing ideal conditions for the development of functional groups such as the aquatic saprophagous and xylosaprophagous syrphid larvae (Howarth and Edmunds 2000; Wolff et al. 2023), the first one being particularly diverse and abundant in our study. Concerning the species and individuals collected, aquatic saprophagous presented significant differences compared to the other larval trophic categories, harboring 71% of the species and 90% of the specimens collected. These results coincide with those reported by Montoya et al. (2021), who obtained higher abundance and richness of flower flies belonging to the genera *Copestylum*, *Palpada* and *Quichuana*, which is a pattern that is consistent with our observation, in which these genera had a good representation in conserved forest i.e., DSF).

In terms of diversity, the DSF (14 exclusive species) and the AFS (12 exclusive species) had the same richness (44 species) and exclusive genera (1). These results differ from those reported by Souza et al. (2014) in Brazil using Malaise traps, where the FE obtained the highest richness (98 species), where more than half the species were exclusive, compared to the more conserved forested areas. However, it should be considered that many Syrphidae species are associated with heterogeneous forests, which seem to offer many microhabitats in European habitats (Branquart and Hemptinne 2000; Meyer et al. 2009). *Argentinomyia* was exclusive to the DSF, a group that has been extensively studied in pristine high Andean forest and Paramo ecosystems in the Tropical Andes, where it harbors high diversity and endemism in highlands (Montoya et al. 2012; Montoya and Wolff 2020; 2023), despite, some species occur in the lowlands close to the Amazonian region (Montoya and Wolff 2023). This is the first report of the genus in the Colombian Amazon region.

Beta diversity of flower flies in the reserve was dominated by species turnover between and within habitats, which coincided with the dissimilarity obtained in the nMDS carried out to compare the species distribution using the Bray–Curtis and Jaccard indices. The nMDS conducted to compare species composition and baits, yielded that decomposing fish was the preferred bait by species of the genera *Copestylum* (most diverse group) and *Ornidia* Lepeletier & Serville, which have been related to decomposing carcasses (Martins et al. 2010; Ramos-Pastrana et al. 2018, 2019). The nMDS also suggests that decomposing shrimp and fermented fruit overlap, which could be a consequence of shared species.

The greatest dissimilarity between DSF and AFS can be attributable to the low number of shared species (*n* = 3). Also, by the plant species that characterize each habitat and the incidence of light, the latter being very important in open areas (AFS and FE), since species occurring in these areas generally do not occur in forests (Gittings et al. 2006). This agrees with the beta diversity results in the pairwise comparison between FE and AFS (*β* = 0.5), which were the least dissimilar, as they are the habitats with the highest number of shared species (*n* = 9), with turnover as the dominant component of beta diversity.

Irvin et al. (1999) state that the seasonal availability of pollen is an important phenological factor, an argument supported by Djellab et al. (2019), who suggest that the availability of food resources during the seasons affects the abundance and population dynamics of flower flies. During our study, greater activity of Syrphids was evidenced during HIR, suggesting that plant heterogeneity, availability of food resources and phytotelmata as main microhabitat are the important phenological factors involved in the composition of flower flies in each habitat and rainy season in the reserve.

### VSRTs as an alternative method to assess Syrphidae diversity in rapid sampling inventories

Our study showed that the VSRTs presented high effectiveness in capturing adult Syrphidae and decomposing fish was the highest efficiency bait, compared to decomposing shrimp and fermented fruits, constituting a potential alternative method for rapid flower fly inventories in the Neotropics. In addition, the similarity observed in NMDS between decomposing shrimp and fermented fruit can be attributed to the shared species compared to decomposed fish. The reserve harbors the endemic Amazonian species such as *Copestylum enriquei*, exclusively collected in the DSF during HIR, suggesting the relevance of VSRTs as a complementary tool for assessing Syrphids diversity.

This work is the first study of flower fly diversity in Colombia using exclusively VSRTs, which showed high sampling integrity. However, the absence of genera and species typical of the Amazonian fauna (Miranda 2017a) evidences the need to use complementary collection methods such as rearing immature, as well as the use of Malaise traps and entomological netting, which allows to cover more groups and make a more compressive diversity estimation. Despite this, the finding of new records and critical functional groups suggest that the VSRTs constitute a potential alternative for rapid inventories of flower flies in the Neotropics. It is noteworthy that in this study the preferred and most effective bait in the collection of Syrphidae was decomposing fish, contributing considerably to the new record of species in Colombia (*n* = 16) and the extension of the range of distribution in the Colombian Amazon (*n* = 31).

## Conclusions

Our results showed that alpha diversity was similar in each habitat, however, the discrimination by the rainy season and bait preference revealed that the patterns of richness and abundance varied considerably, with the high-intensity rainy season being the one that harbored the highest diversity and abundance of the aquatic saprophagous larval trophic category, possibly attributable to the greater availability of water reservoirs (Phytotelmata), which for the sampled sites include two species of Bromeliads, Arecaceae, and Heliconias, as well as one of Musaceae, which potentially harbor relevant and fundamental groups, that play key functions in ecological processes such as decomposition of organic matter and water purification, highlighting the importance of conserving the breeding sites in Nature Reserve of tropical rainforest.

Beta diversity was governed by species turnover in pairwise comparisons between habitats. Given that species turnover is a component related to short seasonal and temporal periods (Noriega et al. 2021), our results suggest that spatially and temporally, each habitat contributes with unique species, providing fundamental functions for environmental sustainability and balance, highlighting the importance of connectivity to ensure diversity and services provision in areas undergoing ecological restoration such as La Avispa Nature and Ecotourism Reserve, in Caquetá, Colombia.

## Supplementary Information

Below is the link to the electronic supplementary material.

Supplementary file1 **Table S1** Main plant composition of the three habitats where adult flower flies (Diptera: Syrphidae) were collected in La Avispa Nature and Ecotourism Reserve, municipality of Florencia, Caquetá, Colombia. (DOCX 13 KB)

Supplementary file2 **Table S2 **Beta diversity of the pairwise comparisons of the three habitats where adult flower flies (Diptera: Syrphidae) were collected in La Avispa Nature and Ecotourism Reserve, municipality of Florencia, Caquetá, Colombia and the values of its two components (turnover and nestedness). (DOCX 13 KB)

Supplementary file3 **Table S3. **Kruskal-Wallis test for baits used in the collection of flower flies (Diptera: Syrphidae) using VSRTs in three habitats of La Avispa Nature and Ecotourism Reserve, municipality of Florencia, Caquetá, Colombia. (DOCX 13 KB)

Supplementary file4 **Fig. S1** Relative abundance and cumulative frequency percentage of adult flower flies (Diptera: Syrphidae) collected in La Avispa Nature and Ecotourism Reserve. Genera of Eristalinae: *Copestylum*,*Ornidia*, *Palpada*, *Quichuana* and *Sterphus*. Genera of Syrphinae: *Argentinomyia*, *Dioprosopa*, *Hybobathus*, *Ocyptamus*,*Pelecinobaccha*, *Relictanum*, *Salpingogaster* and *Toxomerus*. The size of the bars is proportional to the abundance of each species. (TIF 4.46 MB)

Supplementary file5 **Fig. S2 **Comparisons of estimated ^q^D diversity values for communities of adult flower flies (Diptera: Syrphidae) in the three habitats (AFS = Agroforestry System, DSF = Dense Secondary Forest, and FE = Forest Edge) of La Avispa Nature and Ecotourism Reserve, Caquetá, Colombia, for the total sampled period and the two rainy seasons: HIR (high-intensity rainy season) and LIR (low-intensity rainy season). Continuous lines represent interpolation curves, meanwhile dashed lines represent extrapolation curves. Shaded areas represent 95% confidence intervals based on a bootstrap with 200 replicates. a-c. Total sampled, d-f. HIR, g-i. LIR, q0 = observed richness, q1= Shannon's exponential based on shared species and q2 = Simpson's inverse index based on the most abundant species. (TIF 282 MB)

## Data Availability

Data used in the present study are available from the authors upon request.
