# Supplementary material for: Diversity and Baits Preference of Flower Flies (Diptera: Syrphidae) Collected Using Van Someren-Rydon Traps in the Colombian Andean-Amazon Piedmont During Two Rainy Seasons
Source: Neotrop Entomol. 2025 Mar 27;54(1):52. doi: 10.1007/s13744-025-01260-y (PMC11950100; doi:10.1007/s13744-025-01260-y)
Supplement: Supplementary file 1 — Supplementary file1 Table S1 Main plant composition of the three habitats where adult flower flies (Diptera: Syrphidae) were collected in La Avispa Nature and Ecotourism Reserve, municipality of Florencia, Caquetá, Colombia. (DOCX 13 KB) [file 13744_2025_1260_MOESM1_ESM.docx]

**Table S1** Main plant composition of the three habitats where adult flower flies (Diptera: Syrphidae) were collected in La Avispa Nature and Ecotourism Reserve, municipality of Florencia, Caquetá, Colombia.

| Forest Edge | Dense Secondary Forest | Agroforestry System |
| --- | --- | --- |
| Melastomataceae (n = 5)*^a^* | Clyclanthaceae (n = 3) | Araceae (n = 3) |
| Salicaceae (n = 3) | Malvaceae (n = 3) | Arecaecae (n = 2) |
| Fabaceae (n = 2) | Bromeliaceae (n = 2) | Asteraceae (n = 1) |
| Piperaceae (n = 2) | Heliconiaceae (n = 2) | Acanthacae (n = 1) |
| Rubiaceae (n = 2) | Sapindaceae (n = 2) | Euphorbaceae (n = 1) |
| Araceae (n = 1) | Urticaceae (n = 2) | Musaceae (n = 1) |
|  |  | Poaceae (mainly sugarcane*^b^*) |

*^a^*n= Number of species

*^b^*Sugarcane crop (*Saccharum officinarum* L.)
