# Supplementary material for: Diversity and Baits Preference of Flower Flies (Diptera: Syrphidae) Collected Using Van Someren-Rydon Traps in the Colombian Andean-Amazon Piedmont During Two Rainy Seasons
Source: Neotrop Entomol. 2025 Mar 27;54(1):52. doi: 10.1007/s13744-025-01260-y (PMC11950100; doi:10.1007/s13744-025-01260-y)
Supplement: Supplementary file 2 — Supplementary file2 Table S2 Beta diversity of the pairwise comparisons of the three habitats where adult flower flies (Diptera: Syrphidae) were collected in La Avispa Nature and Ecotourism Reserve, municipality of Florencia, Caquetá, Colombia and the values of its two components (turnover and nestedness). (DOCX 13 KB) [file 13744_2025_1260_MOESM2_ESM.docx]

**Table S2** Beta diversity of the pairwise comparisons of the three habitats where adult flower flies (Diptera: Syrphidae) were collected in La Avispa Nature and Ecotourism Reserve, municipality of Florencia, Caquetá, Colombia and the values of its two components (turnover and nestedness).

| Pairs | Turnover | Nestedness | Total dissimilarity |
| --- | --- | --- | --- |
| DSF – AFS | 0.64 | 0 | 0.64 |
| FE – DSF | 0.54 | 0.0076 | 0.55 |
| FE – AFS | 0.49 | 0.0087 | 0.50 |

DSF = Dense Secondary Forest, AFS = Agroforestry System, FE = Forest Edge.
