# Supplementary material for: Diversity and Baits Preference of Flower Flies (Diptera: Syrphidae) Collected Using Van Someren-Rydon Traps in the Colombian Andean-Amazon Piedmont During Two Rainy Seasons
Source: Neotrop Entomol. 2025 Mar 27;54(1):52. doi: 10.1007/s13744-025-01260-y (PMC11950100; doi:10.1007/s13744-025-01260-y)
Supplement: Supplementary file 3 — Supplementary file3 Table S3. Kruskal-Wallis test for baits used in the collection of flower flies (Diptera: Syrphidae) using VSRTs in three habitats of La Avispa Nature and Ecotourism Reserve, municipality of Florencia, Caquetá, Colombia. (DOCX 13 KB) [file 13744_2025_1260_MOESM3_ESM.docx]

**Table S3.** Kruskal-Wallis test for baits used in the collection of flower flies (Diptera: Syrphidae) using VSRTs in three habitats of La Avispa Nature and Ecotourism Reserve, municipality of Florencia, Caquetá, Colombia.

| Baits | Mean | Ranges |  |  |
| --- | --- | --- | --- | --- |
| Fermented banana-pawpaw fruits | 0.94 | 17.00 | A |  |
| Decomposing shrimp | 14.11 | 28.33 |  | B |
| Decomposing fish | 61.56 | 37.17 |  | B |
| *Means with common letters are not significantly different (p > 0.05).* | | | | |
