# Supplementary figures and images for: Diversity and Baits Preference of Flower Flies (Diptera: Syrphidae) Collected Using Van Someren-Rydon Traps in the Colombian Andean-Amazon Piedmont During Two Rainy Seasons
Source: Neotrop Entomol. 2025 Mar 27;54(1):52. doi: 10.1007/s13744-025-01260-y (PMC11950100; doi:10.1007/s13744-025-01260-y)

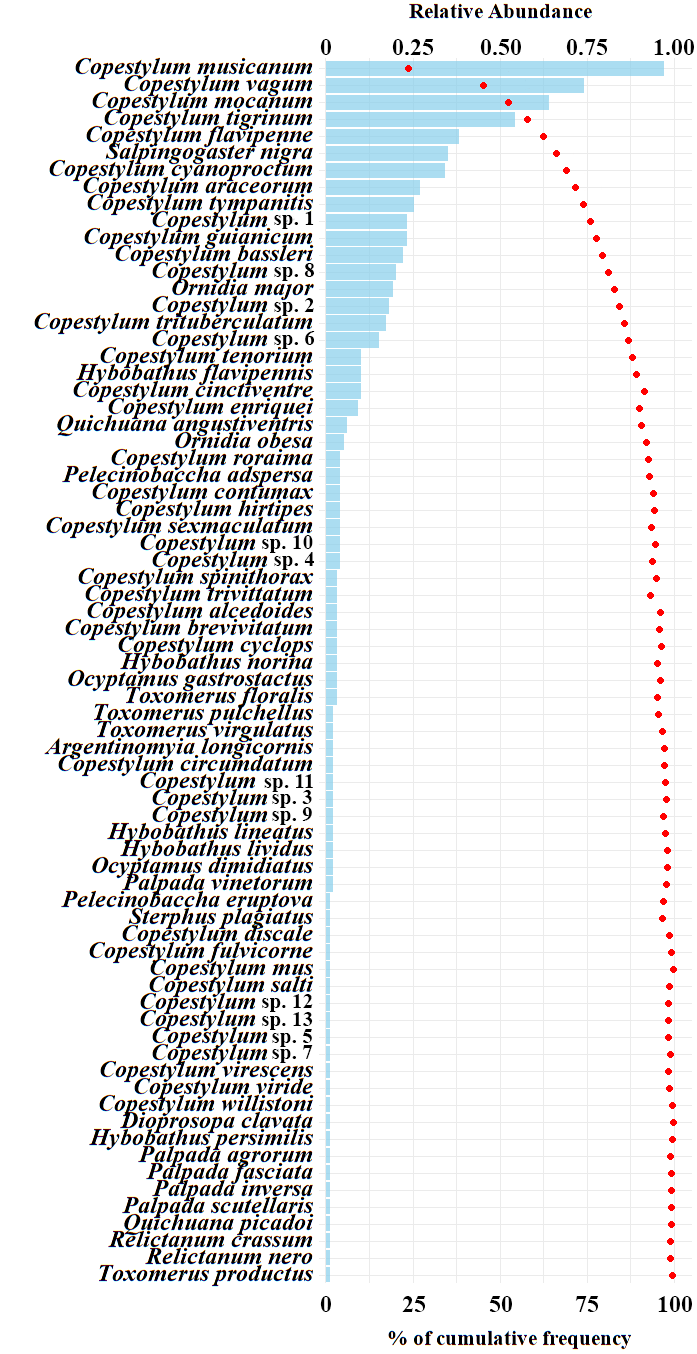

Supplement: Supplementary file 4 — Supplementary file4 Fig. S1 Relative abundance and cumulative frequency percentage of adult flower flies (Diptera: Syrphidae) collected in La Avispa Nature and Ecotourism Reserve. Genera of Eristalinae: Copestylum,Ornidia, Palpada, Quichuana and Sterphus. Genera of Syrphinae: Argentinomyia, Dioprosopa, Hybobathus, Ocyptamus,Pelecinobaccha, Relictanum, Salpingogaster and Toxomerus. The size of the bars is proportional to the abundance of each species. (TIF 4.46 MB) [file 13744_2025_1260_MOESM4_ESM.tif]
